# Tirzepatide, a dual GIP/GLP1-receptor co-agonist preserves cardiac function and improves survival in angiotensin II-induced heart failure model in mice: comparison to liraglutide

**DOI:** 10.1186/s12933-025-02806-5

**Published:** 2025-06-14

**Authors:** Zsombor I. Hegedűs, Márk E. Jakab, Tamás G. Gergely, Nabil V. Sayour, Andrea Kovács, Sára Antal, Tamás Kovács, Péter Ferdinandy, Zoltán V. Varga, Viktória E. Tóth

**Affiliations:** 1 Department of Pharmacology and Pharmacotherapy, Semmelweis University, Budapest, Hungary; 2 Center for Pharmacology and Drug Research and Development, Semmelweis University, Budapest, Hungary; 3 HCEMM-SU Cardiometabolic Immunology Research Group, Budapest, Hungary; 4 MTA-SE Momentum Cardio-Oncology and Cardioimmunology Research Group, Budapest, Hungary; 5 Pharmahungary Group, Szeged, Hungary

**Keywords:** Incretin analogues, Heart failure, Tirzepatide, Liraglutide, HFrEF

## Abstract

**Background:**

Incretin analogues, used for the treatment of type 2 diabetes mellitus and obesity, such as GLP1-receptor agonist liraglutide (Lira) have been shown to reduce major adverse cardiac events in recent clinical trials of heart failure. Tirzepatide (TZP), a dual GIP/GLP1-receptor agonist has shown promising results in the SUMMIT trial as improved cardiovascular outcomes in patients with heart failure with preserved ejection fraction (HFpEF). However, data regarding their use in heart failure with reduced ejection fraction (HFrEF) is lacking. We performed a head-to-head comparative study in a mouse model of non-ischaemic cardiac injury induced by continuous angiotensin II (AngII) infusion, as AngII is a key driver of both heart failure forms.

**Methods:**

Osmotic minipumps were inserted for subcutaneous (s.c.) administration of AngII (1.5 mg/kg/day) in 5-month-old male Balb/c mice or sham surgery was performed. Animals were treated with vehicle (Veh), Lira (300 µg/day i.p.) or TZP (48 µg/day s.c.) for 14 days in the following groups: Sham/Veh (n = 7), AngII/Veh (n = 15), Sham/Lira (n = 7), AngII/Lira (n = 15), Sham/TZP (n = 8), AngII/TZP (n = 15). Cardiac structural, functional and molecular characteristics were assessed by echocardiography, ECG, immunohistochemistry, flow cytometry and qRT-PCR.

**Results:**

Mortality was significantly higher in AngII/Veh animals compared to controls, while AngII/TZP mice showed significantly reduced mortality after 14 days of treatment. Both Lira and TZP caused significant weight reduction compared to controls. AngII given alone also reduced body mass, and this reduction was further enhanced by TZP. Treatment with both compounds preserved cardiac systolic and diastolic function compared with AngII/Veh animals, as shown by normal ejection fraction and E/e’, respectively. Both Lira and TZP decreased the AngII-induced elevation of cardiac fibrosis and hypertrophy markers, including *Ctgf, Col1a1, Col3a1,* and *Nppa,* while TZP also reduced the elevated *Nppb* level. TZP also reduced systemic inflammation, as shown by the reduction in serum CRP levels.

**Conclusions:**

Lira and TZP preserved cardiac function and decreased markers of hypertrophy and fibrosis in mice with AngII-induced heart failure, whereas TZP also significantly decreased mortality. In addition to HFpEF, the use of incretin analogues may also be of clinical relevance in the treatment of HFrEF. However, as patients with heart failure, AngII level is elevated and can cause weight loss/cachexia, the usage of incretin analogues to treat non-obese heart failure patients should be considered.

**Graphical abstract:**

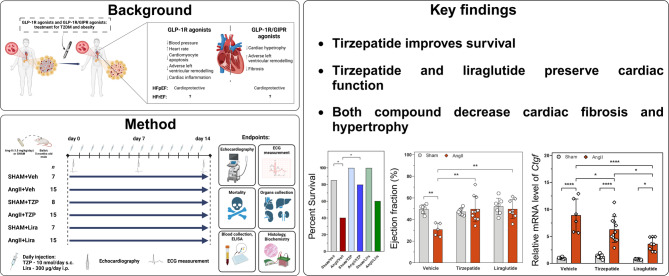

**Supplementary Information:**

The online version contains supplementary material available at 10.1186/s12933-025-02806-5.

## Background

Incretin hormones—glucagon-like peptide 1 (GLP-1) and glucose-dependent insulinotropic polypeptide (GIP)—are secreted by the gastric mucosa to promote post-prandial insulin secretion. Incretin analogues, such as GLP-1 receptor (GLP-1R) agonists (including semaglutide and liraglutide, among others) have gained widespread attention due to their success in the treatment of type 2 diabetes mellitus and obesity. Importantly, semaglutide improved cardiovascular outcomes in the SELECT trial [1], and GLP-1R agonists became cornerstone therapies for reducing risk in patients with atherosclerotic cardiovascular disease (ASCVD) [2].

While the clinical use and potential indications of incretin analogues are increasing rapidly, many questions remain unanswered regarding their effects on the cardiovascular system, especially in heart failure [3]. In patients with heart failure with preserved ejection fraction (HFpEF) and obesity, semaglutide decreased heart failure-related symptoms and improved quality of life in patients with or without type 2 diabetes (STEP-HFpEF [4] and STEP-HFpEF DM [5] trials), however, the studies did not provide evidence regarding cardiovascular outcomes. Moreover, in patients with heart failure with reduced ejection fraction (HFrEF), two clinical studies of liraglutide (FIGHT [6] and LIVE [7] trials) have shown no beneficial effects in heart failure-related outcomes or improvement in left ventricular ejection fraction, whereas even potential harm has been suggested regarding an increased risk of heart failure hospitalisations [8, 9]. Thus, the use of incretin analogous in heart failure is not fully established currently [3, 10].

As the field of incretin analogues is expanding rapidly, novel therapeutic options are arising. Tirzepatide, a novel dual incretin analogue targeting both GLP-1R and GIP receptor (GIPR), has been approved for the treatment of type 2 diabetes and obesity. The recent SUMMIT trial investigating tirzepatide therapy in patients with HFpEF provided the first evidence of an improved cardiovascular outcome (a composite of death from cardiovascular causes or worsening heart failure)[11]. Nevertheless, data regarding the effect of tirzepatide in heart failure across the full spectrum of ejection fraction is currently lacking.

Moreover, the mechanisms behind the beneficial cardiovascular effects of incretin analogues are not completely understood and may include effects beyond weight loss, e.g. related to decreased systemic inflammation [12] and immunomodulatory effect [13], alleviated cardiac adverse remodelling [14], and increased endocardial GLP-1R signalling [15]. Further studies investigating the cardiac effects of incretin analogues and comparison of GLP-1R agonists and dual GLP-1R/GIPR agonists are needed to better understand the potential benefits and risks of incretin analogue therapy in heart failure.

In this study, we carried out an angiotensin II-induced cardiac injury model in mice leading to reduced left ventricular ejection fraction, to perform a head-to-head comparison of the effects of GLP-1R agonist liraglutide, and GLP-1R/GIPR dual agonist tirzepatide on mortality and cardiac functional and structural changes. Furthermore, we investigated the potential mechanisms behind their cardiovascular effects.

## Methods

### Experimental animals

All procedures were approved by the National Scientific Ethical Committee on Animal Experimentation and the Semmelweis University's Institutional Animal Care and Use Committee (H-1089 Budapest, Hungary) in accordance with NIH guidelines (National Research Council [2011], Guide for the Care and Use of Laboratory Animals: Eighth Edition) and permitted by the government of Food Chain Safety and Animal Health Directorate of the Government Office for Pest County (project identification code: PE/EA/01259-6/2022; date of approval: December 2022).

3-month-old male Balb/c mice were ordered from Charles River Laboratories, started weight of the animal was between 22 and 29 g. Animals were grown for 2 months in individually ventilated cages (IVC) under standard conditions (23 ± 2 °C, 12-h light/dark cycles and 35–75% relative humidity) with free access to standard rodent chow and tap water ad libitum. Three or four animals were kept in cages with environmental enrichment elements. The study started when the animals reached the 5 month old age with a weight of 24–32 g.

### Experimental design of angiotensin II-induced cardiac injury model

At the start time of the study baseline echocardiography and body weight measurement were carried out. Animals were randomly assigned to the following six groups: angiotensin II-induced (AngII) or sham-operated (Sham), with a treatment of vehicle (Veh), tirzepatide (TZP) or liraglutide (Lira). Cardiac injury was induced by continuous angiotensin II infusion (dissolved in physiological saline, Bio-Techne R&D Systems Inc., Minneapolis, MN, USA) with a dose of 1.5 mg/kg/day. The AngII induction was carried out with subcutaneously inserted osmotic minipumps (Alzet, Cupertino, CA, USA), as described previously [16]. Briefly, the animals were anaesthetized with isoflurane, for induction 5% V/V in 100% O_2_ was used in the induction chamber and for maintenance, the animal received 2% V/V isoflurane via a nose cone. During the anaesthesia, animals breathed spontaneously, the pain reflex was tested by pinching the toes every 10 min, and the core temperature of the animals was monitored with a rectal probe and maintained at 37 ± 0.5 °C using a heating pad. The osmotic minipump implantation was carried out under aseptic conditions. Hair was removed, an incision was made on the right middle region of the back, and an atraumatic scissor was used to make a subcutaneous pouch for the osmotic minipump. The wound was closed by suturing, disinfection was carried out on the surgical area and for pain relief tramadol (Contramal® 50mg/ml injection, 00927R, Stada Arzneimittel AG, Bad Vilbel, Germany) was administered (10 mg/kg in 0.2 ml, dissolved in physiological saline) subcutaneously before the animals awakened. Animals went through daily inspections to determine the need for further analgesia. In the control groups sham operation was carried out to ensure the same stress factors as in the AngII-induced groups.

The animals were treated with incretins for 14 days, starting on the day of the surgery. Based on the treatment, AngII-induced and sham-operated animals were separated into 3 groups: (i) vehicle groups (Sham/Veh n = 7 and AngII/Veh n = 15) received physiological saline intraperitoneally (i.p.) on a daily basis in an equivalent volume relative to the body weight. (ii) tirzepatide groups (Sham/TZP n = 8 and AngII/TZP n = 15) received tirzepatide (TZP, dissolved in physiological saline, BOC Sciences, London, UK) subcutaneously (s.c.) with a dose of 48 µg/kg/day [17] (iii) liraglutide groups (Sham/Lira n = 7 and AngII/Lira n = 15) received liraglutide (Lira, (Victoza®, Solution for injection in 6 mg/ml pre-filled pen, NS6JE85, NOVO NORDISK, Denmark) via intraperitoneal (i.p.) injection with a 300 µg/kg/day dose [18]. Daily weight measurement was carried out to follow the weight changes of the animals due to GLP-1 analogues treatment. On the 15th days after the surgery the animals were euthanized in a humane way using pentobarbital (90 mg/kg) and a whole-body perfusion was carried out with phosphate-buffered saline (PBS). During the sacrifice of the mice, blood collection was carried out and serum samples were isolated and stored for further characterization. Organs were harvested for further histological and biochemical characterization.

### Echocardiography

Echocardiography was performed to characterize the functional and structural changes of the heart due to the angiotensin II induction and to characterize the effects of the GLP-1 analogues on the heart. Baseline echocardiography was performed before the surgery. The follow-up echocardiographic measurements were carried out on the seventh and fourteenth day after the surgery, as described earlier [19].

Briefly, induction of the animals’ anaesthetization was carried out with 3% V/V isoflurane in 100% O_2_, and for maintenance, 1.5% V/V isoflurane in 100% O_2_ was used through a nose cone. During the anaesthesia, animals breathed spontaneously, the pain reflex was tested by pinching the toes every 5 min, and the core temperature of the animals was monitored with a rectal probe and maintained at 37 ± 0.5 °C using a heating pad. At the time of the measurement, respiratory rate and heart rate were monitored. Echocardiographic analysis was performed using the Vevo 3100 high-resolution in-vivo imaging system (Fujifilm VisualSonics, Toronto, Canada) with an ultrahigh-frequency MX400 transducer (30 MHz, 55 frames per second). After chest hair removal, two-dimensional cines were obtained from the long-axis, short-axis and apical four-chamber views of the heart. Short-axis recordings at the mid-papillary muscle level using M-mode were obtained to measure various parameters, including left ventricular internal diameter in systole (LVIDs) and diastole (LVIDd), as well as the thickness of the left ventricular anterior and posterior walls. End-diastolic and end-systolic left ventricular areas were assessed from both short- and long-axis two-dimensional B-mode images. Diastolic parameters were measured from the apical four-chamber view. Additionally, pulse-wave Doppler and tissue Doppler techniques were utilized to assess early mitral inflow velocity and early diastolic velocity of the mitral annulus (e’), respectively.

Echocardiographic recordings were analysed in a blinded method using the VevoLab 5.9.0 software (Fujifilm VisualSonics, Toronto, Canada). The ejection fraction (EF) was calculated as (SV/LVEDV) *100, where SV is stroke volume and LVEDV is end-diastolic volume. The end-diastolic and end-systolic volume (LVESV) parameters were determined from the rotational volumes of the left ventricular trace at diastole and systole, based on the long axis line of the spline. Stroke volume was calculated using the LVEDV—LVESV equation and [(LVIDd—LVIDs)/LVIDd] *100 was used to determine the fractional shortening (FS). Cardiac output (CO) calculation was carried out following the (SV * HR/1000) equation. To determine the LV mass a modified cubic formula for rodents was used (LV mass = 1.04*[(LVIDd + LVAWd + LVPWd)^3^—LVIDd^3^] * 0.8 + 0.6). Relative wall thickness (RWT) was calculated as 2*LVPWTd/LVIDd, and left ventricular remodelling index (LVRi) was calculated as LV mass/LVIDd [20, 21]. Due to the large body weight difference between the groups, several parameters were normalized to body surface area (BSA), such as CO, SV, volumes of the left ventricle in systole and diastole and the dimensions of the wall. BSA was determined following the 9.8*BW^2/3^ equation. The cardiac index was calculated following the CO/BSA Eq. (21).

### Electrocardiography (ECG)

Simultaneously with echocardiography, electrocardiography measurements were performed before the treatment, and on the seventh and fourteenth day. Animals were under anaesthesia as mentioned above. The main three leads of ECG were placed (I., II. and III.) in 1 min period of each. Subcutaneous electrodes were used for the registration. The evaluation of the records was carried out using the LabChart 8 Reader software (ADInstruments, Oxford, UK) in a blinded manner. Heart rate was measured in all leads and an average was calculated. Lead II was used to determine the length of the PQ and QRS complex in time and the amplitude of the QRS complex.

### Histological analysis

During the sacrifice of the animal basal parts of the heart were harvested for histological investigation after whole-body perfusion. Neutral buffered formalin was used for 24 h to fix the cardiac tissue, after fixation dehydration was performed with a series of upscale alcohol solutions and the tissue was embedded in paraffin. Four-micrometre-thick sections were used for histological and immunohistological characterization. Leica LMD6 microscope (Wetzlar, Germany) was used to visualize and capture all staining.

#### Haematoxylin–eosin staining

Haematoxylin–eosin staining was used to investigate the morphologic and pathologic alterations of the cardiac tissue. The paraffin-embedded heart sections were de-paraffinized and hydrated, followed by haematoxylin staining and counterstained with eosin.

#### Picrosirius red staining

Pre-treatment with 0.2% aqueous phosphomolybdic acid was performed after initial de-paraffinization and hydration. Then sections were stained with 0.0125% picrosirius red for 1 h at room temperature. 0.01 N HCl was used for the washing step. The size of the cardiac fibrosis was assessed and quantified by ImageJ software.

### Immunohistochemistry (IHC)

After de-paraffinization and hydration, the paraffin-embedded heart sections underwent antigen retrieval (citrate buffer pH = 6 or Tris buffer pH = 9) for 15 min. 3% H_2_O_2_ was used to block endogenous peroxidase, followed by 2.5% goat or horse serum and 2% milk powder or bovine serum albumin blocking. Sections were incubated with primary antibodies (Iba1 (Fujifilm, Neuss, Germany), CD45 (abcam, Cambridge, UK) and CD3 (Cell Signaling Technology, Danvers, USA)) overnight at 4 °C. After three wash steps with PBS, secondary antibody incubation was performed with anti-rabbit IgG HRP (Cell Signaling Technology, Danvers, USA) or anti-goat IgG HRP (Vector Laboratories, Newark, USA). The last wash step was followed by diaminobenzidine incubation for signal amplification. Percent of positive areas was assessed and quantified by ImageJ software.

### Lectin histochemistry

For further characterization of cardiac hypertrophy and fibrosis, lectin histochemistry was carried out. After 30 min antigen retrieval (Antigen Unmasking Solution, Tris-based, pH = 9, Vector Laboratories, Newark, CA, USA), sections were incubated overnight at 4°C with wheat germ agglutinin (WGA-FITC, 1:50, Sigma Aldrich, L4895) and isolectin B4 (ILB4-DyLight 594, 1:50, Invitrogen, L32473). The cross-sectional area of the heart was captured and the previously published CardiLect protocol [22] was used for the analysis. At least 6 images per heart were analysed. Microvascular density was determined by calculating the ratio of the capillary count to the average cross-sectional area of the cardiomyocytes.

### Enzyme-linked immunosorbent assay (ELISA)

A mouse C-Reactive Protein/CRP ELISA kit (Bio-Techne R&D Systems Inc., Minneapolis, MN, USA) was used to determine the circulating CRP in the serum of the mice. The experiments were performed according to the manufacturer's protocol.

### RNA isolation and quantitative reverse transcription-polymerase chain reaction (qRT-PCR)

The chloroform/isopropanol precipitation method was used for total RNA isolation from the heart tissue of the mice as previously described [23]. The homogenization was carried out with TissueLyser (Qiagen, Venlo, the Netherlands). Qiazol® (Qiagen, Venlo the Netherlands) was added to each sample as lysis buffer and after two rounds of 5-min homogenization the samples were centrifuged. From the supernatant DNA and protein were precipitated with chloroform. After centrifugation total RNA was precipitated using isopropanol. 75% ethanol (VWR, Radnor, PA, USA) was used to wash the pellet four times, followed by resuspension of the total RNA in nuclease-free water. RNA concentration was measured by spectrophotometry with Nanophotometer® N60, (Implen, München, Germany).

For cDNA synthesis, 1 µg of total RNA was used and performed by Sensifast cDNA synthesis kit (Bioline, UK) according to the manufacturer’s protocol. A 20-fold dilution was prepared in RNAse-free water from each sample. qRT-PCR reactions were performed on a LightCycler® 480 II instrument (Roche, Germany) by using Sensifast SYBR Green master mix (Bioline, UK).

Sequences of primers are shown in Table S2. Ribosomal protein L13a (*Rpl13a*) was used as housekeeping gene. Results were calculated with 2^−ΔΔCp^ evaluation method.

### Flow cytometry measurements

LEGENDplex™ mouse anti-virus response panel was purchased from BioLegend (CA, USA) to investigate the level of circulating interferons and other key proinflammatory cytokines in the serum of the animals. The experiment was carried out according to the manufacturer’s protocol. Briefly, a filter plate was used for the measurement, the plate was pre-wetted with 1X wash buffer. A twofold dilution from the serum samples was made in assay buffer, the standards were diluted in matrix A solution. After the samples and standards were loaded to the plate, the beads mixture was added to each well and incubated for 2 h at room temperature. Then the plate was washed with 1X wash buffer and the detection antibodies were added to each well, and incubated for 1 h at RT. Streptavidin–phycoerythrin (SA-PE) solution was loaded to each well directly and incubated for 30 min. After a washing step, the beads were resuspended in 1X wash buffer. A Cytek® Northern Lights™ (NL)-CLC flow cytometer (Cytek Biosciences Inc, CA, USA) was used for data acquisition. Data analysis was carried out by a blinded method using the LEGENDplex™ qognit cloud-based software and manually in GraphPad Prism software (version 8.0.1).

### Statistical analysis

All measurements and evaluations were performed in a blinded manner. All data were obtained from a minimum of five independent measurements, with each data point representing values derived from individual experimental animals. The results are expressed as the mean ± standard deviation (SD). GraphPad Prism software (version 8.0.1) was used to perform statistical analysis, the p-value of < 0.05 was considered statistically significant for all comparisons. The normality of the data was tested using the Shapiro–Wilk test. Survival curves were generated using the Kaplan–Meier method and comparisons between survival curves were conducted using the log-rank Mantel-Cox test. Outliers were identified by ROUT analysis with a Q value of 5%. Two-way ANOVA followed by Holm–Sidak post hoc test was used for multiple comparisons between related groups.

## Results

### Tirzepatide treatment attenuates the mortality associated with angiotensin II-induced cardiac injury

**Fig. 1:**
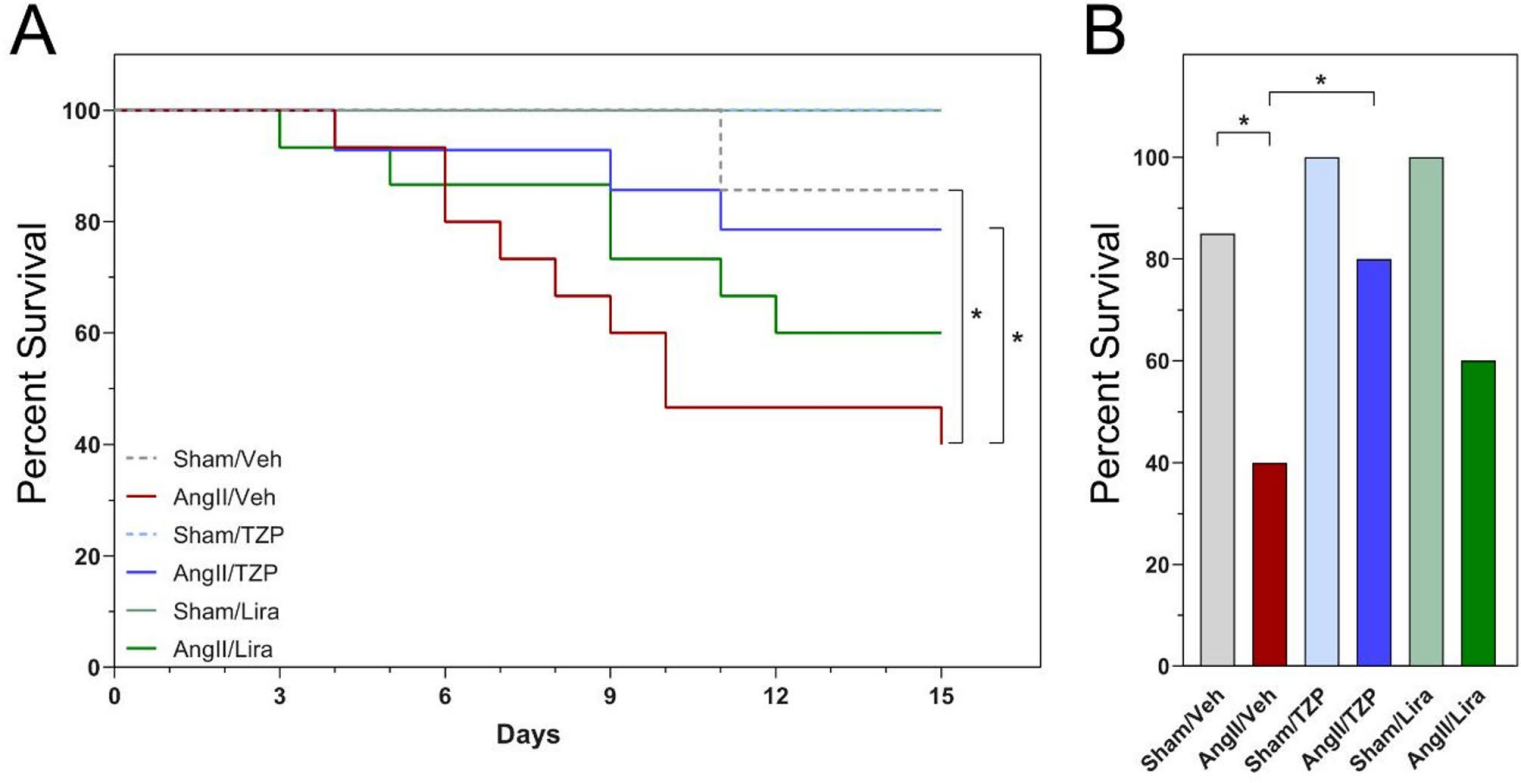
Tirzepatide significantly reduced the mortality of angiotensin II infusion. Percent of survival animals during the follow-up period **A** and on the day of termination **B** Kaplan–Meier method, log-rank Mantel–Cox test, *P < 0.05

Comparing the Sham-operated and AngII-induced groups during the 14 days of follow-up, mortality was significantly higher in the vehicle-treated groups with AngII-infusion (Fig. 1A). The highest mortality was observed in the AngII/Veh group, where nine animals died (60%) during the 14-day follow-up period. In contrast, in the AngII/Lira group, six animals died (40%), while in the AngII/TZP group, mortality was limited to three mice (20%) (Fig. 1A). A significant reduction was observed between the vehicle and TZP-treated group following angiotensin II induction (P = 0.038, log-rank Mantel–Cox test). During the follow-up, 1 animal died in the Sham/Veh group, while in the case of the Sham/TZP and Sham/Lira groups, there was no mortality. Based on these results, TZP significantly increased the survival chance of the animals in the Ang II-induced cardiac injury model (Fig. 1B).

### Treatment with GLP-1 analogues led to significant reduction in body weight

**Fig. 2:**
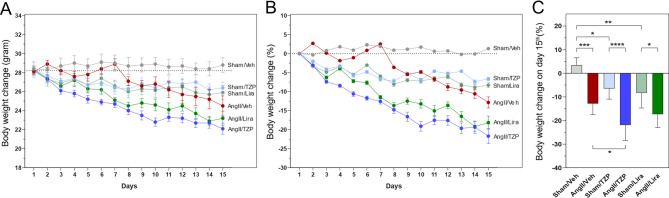
Tirzepatide and liraglutide treatment led to robust weight reduction combined with AngII. Body weight changes during the treatment period in grams **A**, Mean ± SEM) and in percent **B**, and on the day of termination **C**. Two-way ANOVA followed by Holm–Sidak post-hoc test, *P < 0.05, **P < 0.01, ***P < 0.001 and ****P < 0.0001

The weight of the animals was registered daily during the follow-up period. Significant weight reduction was seen with both TZP and Lira treatment, in accordance with literature data (Fig. 2A, [24, 25]). In the Sham-operated groups, treatment with TZP and Lira led to a significantly reduced body weight (TZP—6.7%, from 28.3 ± 2.3 g to 26.4 ± 1.7 g; Lira—8.3%, from 28.2 ± 1.4 g to 25.9 ± 2.0 g) respectively, compared to the vehicle-treated group, where a 1.3% weight gain (from 28.4 ± 2.0 to 28.8 ± 1.9 g) was observed by the end of the two-week treatment period. A 12.8% reduction (from 28.1 ± 1.1 to 24.5 ± 2.8 g) in body weight was observed after two weeks of AngII-infusion compared to the Sham-operated animals. Moreover, treatment with both agents caused a significant weight reduction in the AngII-induced groups (TZP: from 28.2 ± 1.2 to 22.1 ± 2.0 g, Lira: from 28.3 ± 1.4 to 23.2 ± 2.2 g) compared to their respective Sham-operated groups, whereas treatment with tirzepatide further enhanced the weight loss in the AngII-treated group as well (Fig. 2B).

### Tirzepatide preserved cardiac function following one week of continuous angiotensin II induction

To characterize the effects of tirzepatide and liraglutide on angiotensin II-induced cardiac morpho-functional changes, echocardiography was performed at baseline, after one week of continuous AngII infusion and before termination at week of the treatments.

**Fig. 3:**
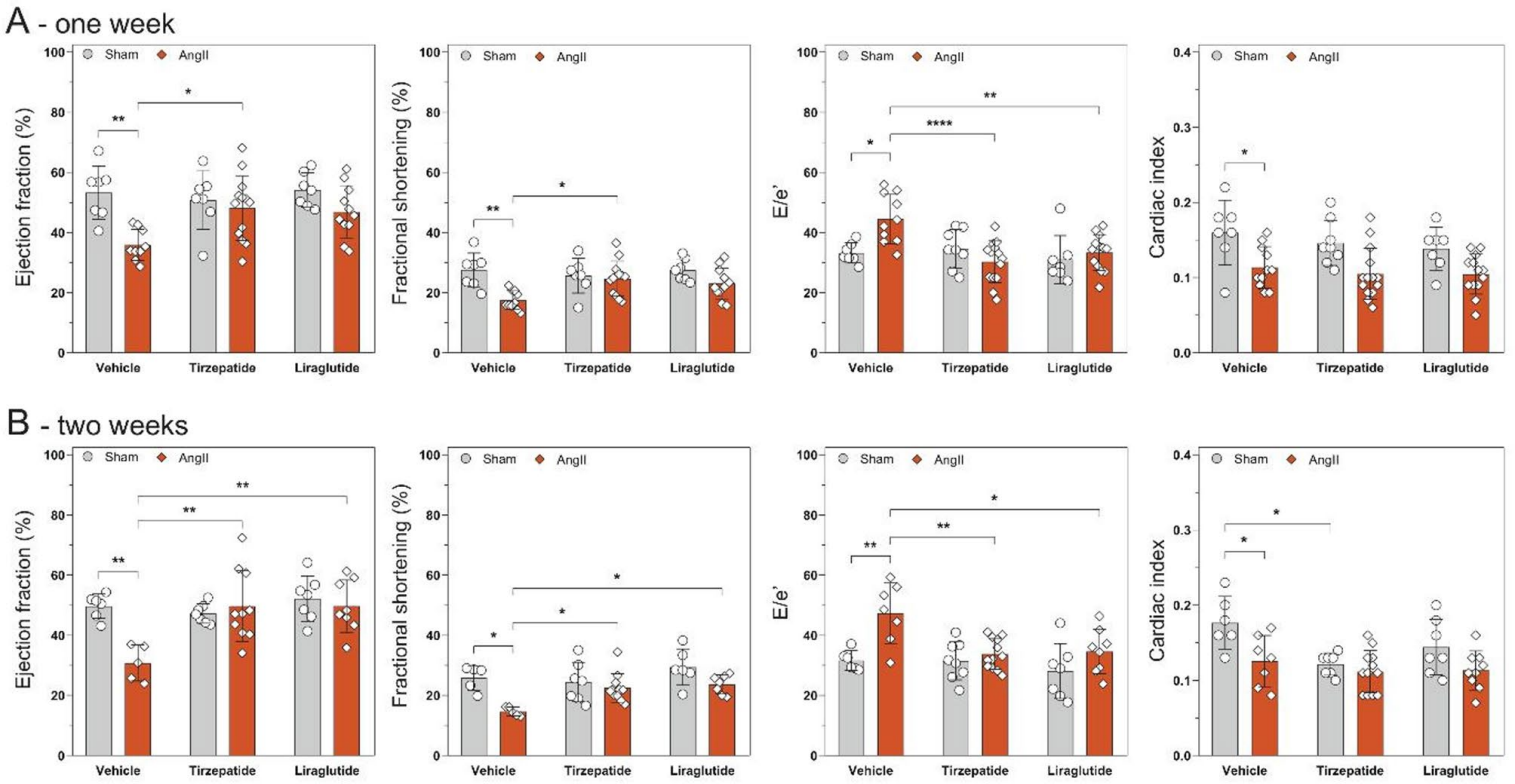
Tirzepatide and liraglutide preserved cardiac function. Functional parameters of the heart after one **A** and two weeks **B** of AngII infusion. Two-way ANOVA followed by Holm–Sidak post-hoc test, *P < 0.05, **P < 0.01, and ****P < 0.0001

AngII alone led to a significant reduction in both systolic (i.e. EF, FS and CI) and diastolic (i.e. E/e’) function of the heart at all time points, compared to the Sham group (Fig. 3), whereas two week long treatment with TZP and Lira preserved cardiac function after AngII infusion (Fig. 3). In addition, tirzepatide showed preservation of systolic cardiac function at the earlier one-week follow-up time point as well.

### Angiotensin II infusion leads to cardiac structural remodelling, which is reduced by treatment of both drugs

**Fig. 4:**
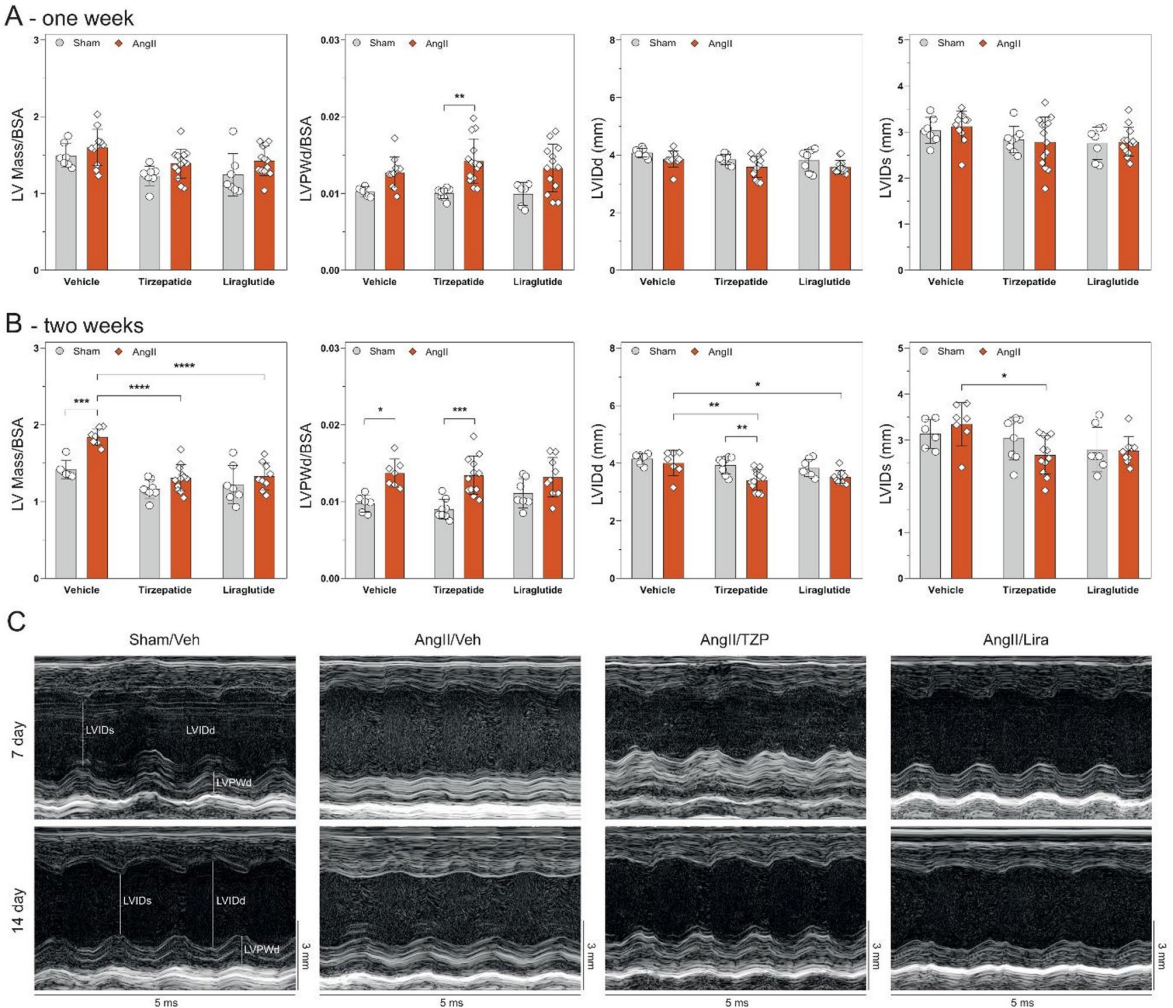
Both drugs attenuate the extent of remodelling caused by angiotensin II infusion. Structural changes of the heart at one week **A** and two weeks **B** Continuous AngII infusion led to the remodelling of the left ventricle. Representatives **C** from the long-axis view M-mode of the heart show the wall thickness increase due to AngII infusion and treatment with TZP. Two-way ANOVA followed by Holm–Sidak post-hoc test, *P < 0.05, **P < 0.01, ***P < 0.001 and ****P < 0.0001

Continuous infusion of angiotensin II for 14 days induced morphological changes in the left ventricle. The mass of the left ventricle (LV mass) and parameters of ventricular dimensions (i.e. LVPWd) were normalized to the body surface area (BSA) of the animals, as there were differences in body weight between the groups due to the treatments. After one week of follow-up, AngII failed to induce cardiac hypertrophy (Fig. 4A). At the end of the follow-up period, the mass of the left ventricle and LVPWd was significantly increased due to AngII induction compared to Sham-operated controls. Treatment with TZP or Lira in the AngII-infusion groups prevented the cardiac mass increase, however, the increase in LVPWd was not affected by treatment with the incretin analogues. Interestingly, left ventricular dilation was not seen with AngII-infusion. In the AngII-induced groups, TZP caused a significant reduction in diameter at systole and diastole compared to AngII infusion alone. Representatives were captured from the long axis M-mode to show the structural changes of the left ventricle during the follow-up period (Fig. 4C). Additional parameters of echocardiography are available in Table S1.

### Electrophysiological changes related to hypertrophic remodelling were attenuated by TZP and lira treatment

**Fig. 5:**
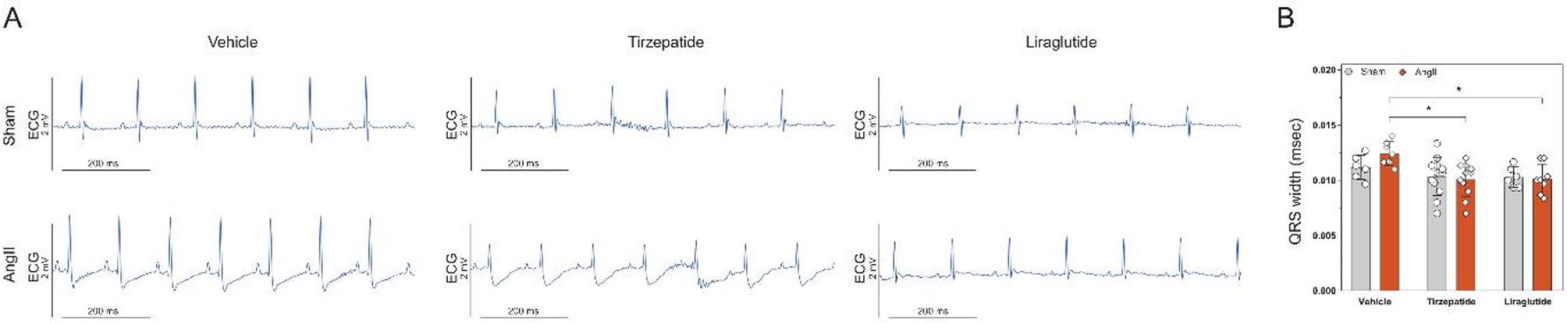
Angiotensin II produced conduction and repolarization abnormalities, mitigated by tirzepatide and liraglutide. Representative electrocardiograms from lead II **A** and QRS width measured in lead III **B** Two-way ANOVA followed by Holm–Sidak post-hoc test, *P < 0.05

ECG analysis identified important ventricular conduction and repolarization abnormalities in AngII-treated animals, consistent with myocardial structural changes due to hypertrophic remodelling. These included a prolonged QRS duration, a distinctly negative JT segment, and fused or inseparable J and T waves. By the end of the two-week treatment period, QRS widening indicated ventricular conduction delays, a hallmark of heart failure-related electrical dysfunction. However, both Lira and TZP attenuated this effect (Fig. 5).

AngII infusion also led to a significant heart rate increase during the first week and at the end of the 14-day treatment in vehicle-treated animals (see Supplementary Table 4.). In contrast, Lira- and TZP-treated groups did not exhibit this tachycardic response, suggesting a reduced need for chronotropic adaptation to the hemodynamic stress induced by AngII.

### Tirzepatide and liraglutide treatment reduced the angiotensin II-induced cardiac fibrosis and hypertrophy

Histological and qRT-PCR characterization were performed to investigate the pathological changes related to AngII-induced cardiac remodelling.

**Fig. 6:**
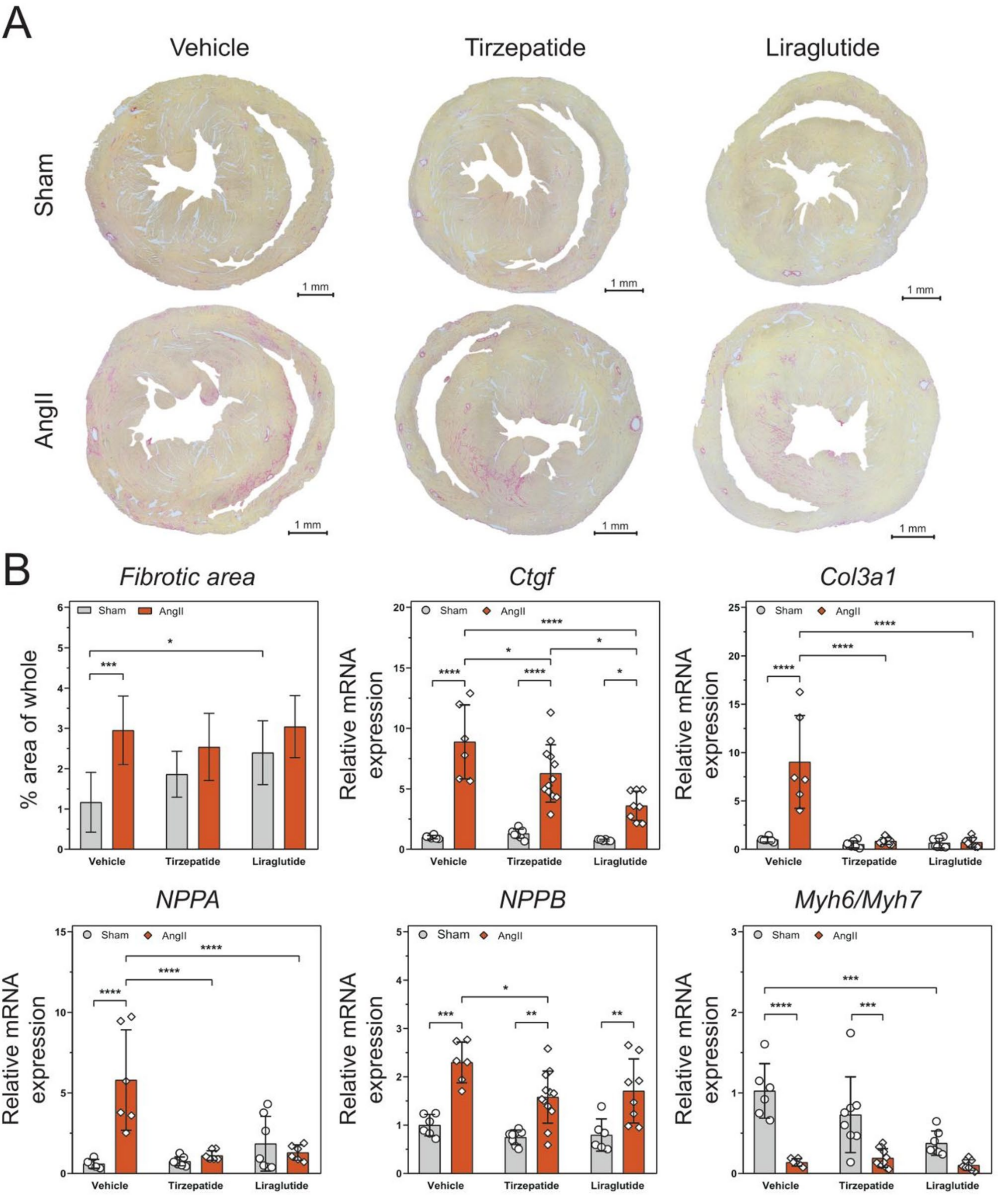
Tirzepatide and liraglutide alleviate the fibrosis and hypertrophy caused by angiotensin II infusion. Representatives of Sirius red staining in the heart **A** show that AngII infusion led to higher fibrosis compared to the controls. Markers of fibrosis and hypertrophy **B** Two-way ANOVA followed by Holm–Sidak post-hoc test, *P < 0.05, **P < 0.01, ***P < 0.001 and ****P < 0.0001

Increased fibrosis was observed in cardiac tissue due to AngII induction alone (Fig. 6A) AngII increased the expression level of *Ctgf* and *Col3a1* as markers of fibrosis, compared to Sham-operated animals, whereas TZP and Lira significantly decreased the expression level of these markers in the AngII-induced groups (Fig. 6B).

Gene expression of cardiac hypertrophy markers was examined by qRT-PCR. *Nppa* and *Nppb* significantly increased due to AngII, compared to the Sham-operated group. TZP and Lira were able to maintain the normal expression level of *Nppa*, however, significant differences were observed between the AngII-induced and Sham-operated groups (Fig. 6B). *Myh6* and *Myh7* are genes that encode the α- and β-myosin heavy chain (MHC), respectively, and can serve as a marker for heart failure-related sarcomeric remodelling. In healthy rodents, the αMHC isoform (encoded by *Myh6*) is the dominant isoform, and *Myh6* expression is reduced due to cardiac stress, while the expression of *Myh7* is upregulated. These changes induce a diminution in the ratio of *Myh6/Myh7*, which indicates cardiac remodelling. In our experiment, changes in the ratio of the expression of *Myh6* and *Myh7* were observed. Significant decrease occurred in the case of the vehicle and TZP between AngII-induced and Sham-operated groups. Interestingly, Lira treatment without AngII infusion also mildly decreased this ratio.

**Fig. 7:**
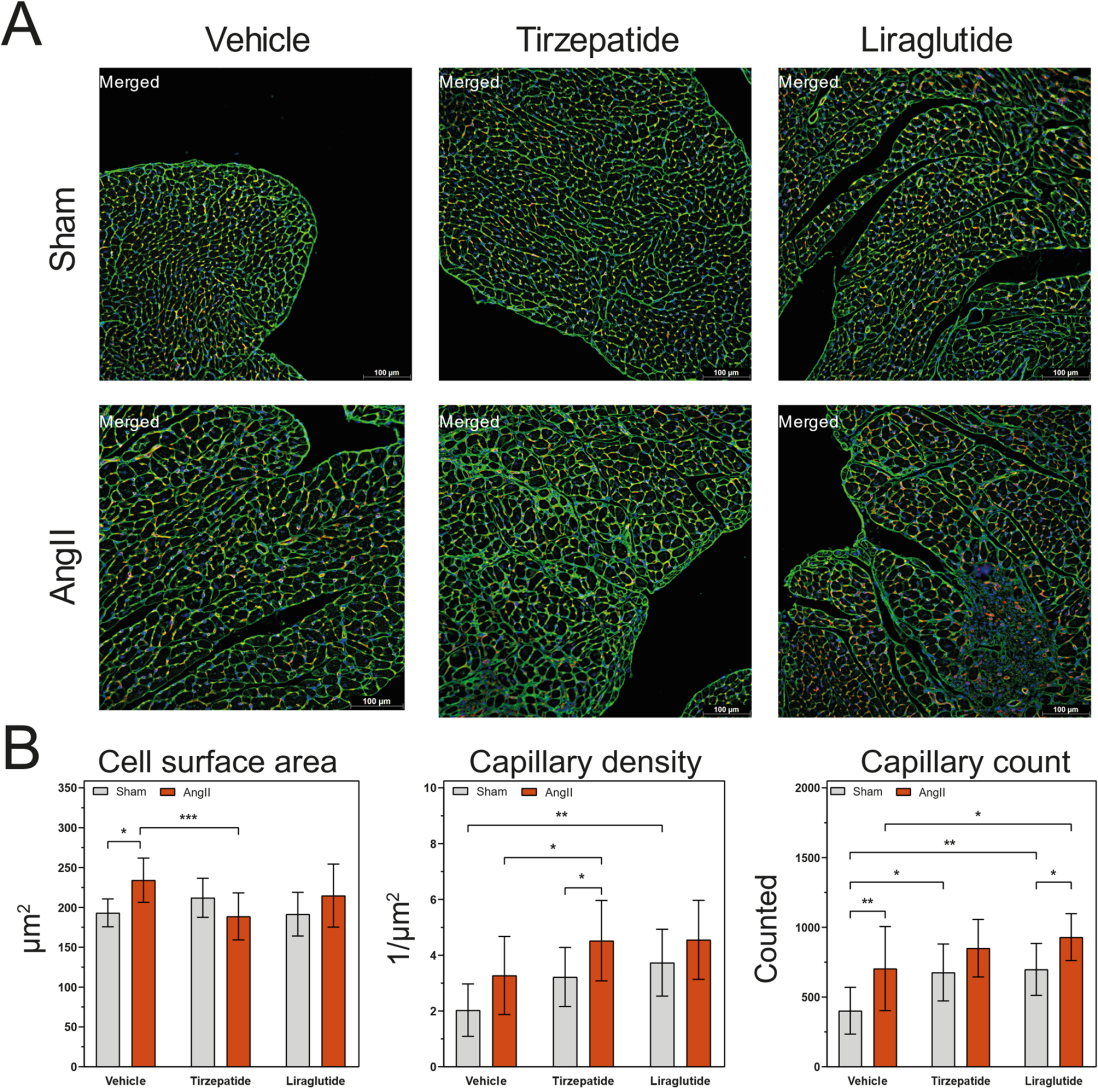
Tirzepatide reduces hypertrophy and increases capillary density of the heart in angiotensin II-induced cardiac injury. Representatives of lectin histochemistry from the heart cross-sectional area **A** cell surface area (CSA), capillary density, and capillary count were calculated from the captured pictures with ImageJ **B** Two-way ANOVA followed by Holm–Sidak post-hoc test, *P < 0.05, **P < 0.01, ***P < 0.001

For further characterization of cardiac hypertrophy, lectin histochemistry was performed to determine the cell surface area of the cardiomyocytes and the capillary density. CardiLect protocol [22] was used for the stainings and for the analysis of the captured images (Fig. 7A). AngII infusion alone caused a significant increase in cell surface area, which was reduced by TZP treatment. Comparing the two treatments to each other, TZP showed a significant reduction next to Ang II infusion (Fig. 7B). Combining TZP treatment with AngII leads to a significant increase in capillary density compared to Sham/TZP and AngII/Veh groups. Interestingly, in Sham-operated groups, Lira treatments caused a significant increase in capillary density compared to the control (Fig. 7B).

### Tirzepatide attenuates mild systemic inflammation caused by angiotensin II infusion

**Fig. 8:**
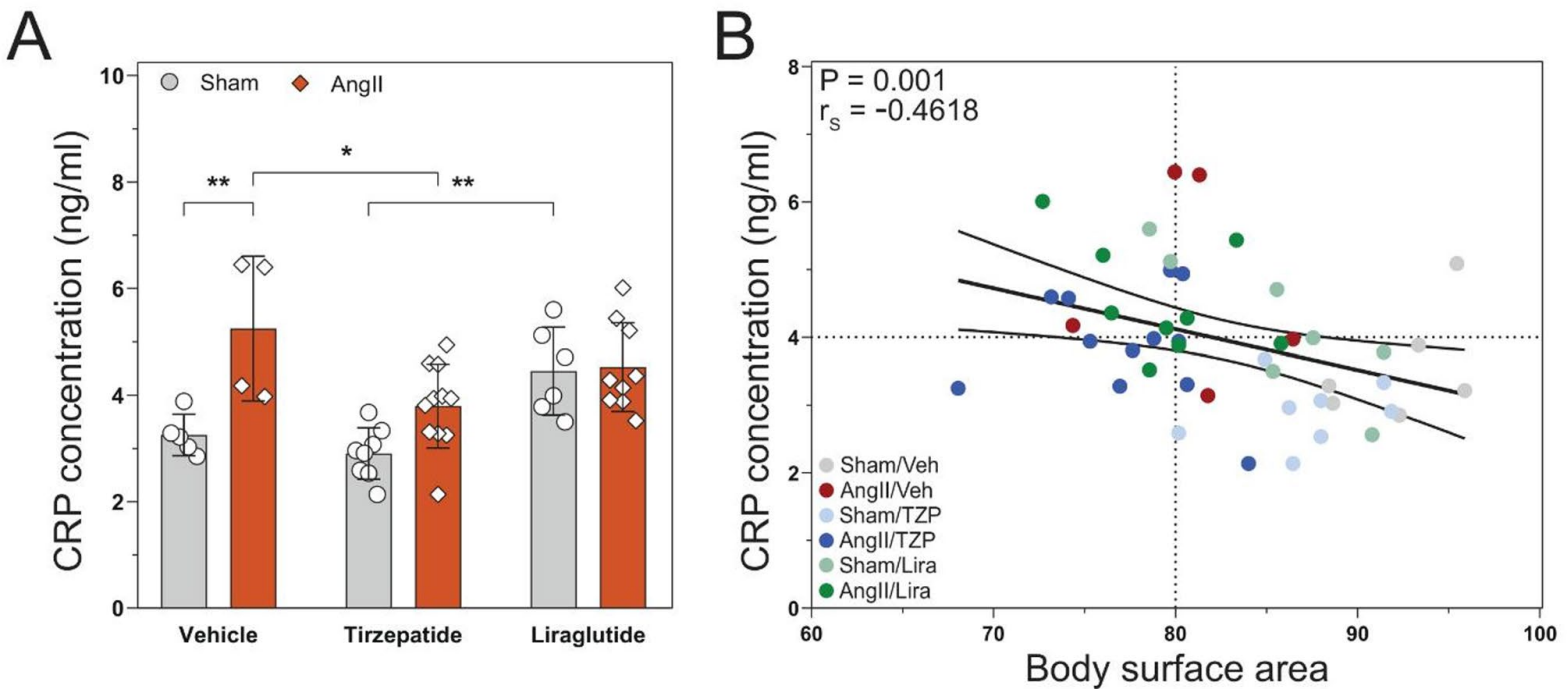
Angiotensin II infusion led to a mild-systemic inflammation, which was reduced by tirzepatide. C-reaction protein (CRP) concentration in the serum **A** Correlation analysis **B** demonstrates a negative correlation between CRP level and body surface area (BSA). Two-way ANOVA followed by Holm–Sidak post-hoc test, *P < 0.05, **P < 0.01, Spearman’s correlation test, **P < 0.0021

The concentration of C-reactive protein (CRP) and several cytokines and chemokines were measured from serum samples. CRP has shown significant elevation in the AngII/Veh compared to its control (P = 0.007 vs Sham/Veh) and treatment with TZP reduced this increase to the normal level (P = 0.03 vs AngII/Veh, Fig. 8A). A negative correlation was observed between the level of CRP and body surface area (BSA) of the mice (P = 0.001). Figure 8B shows that although TZP treatment combined with AngII infusion decreased the BSA, did not increase the concentration of CRP in the system. Meanwhile, the level of circulating cytokines and chemokines, such as *TNFα, IFNα and γ, IL-6, CCL2*, and *CXCL1* did not show any changes due to the AngII induction or the treatments (Suppl. Table S3).

**Fig. 9:**
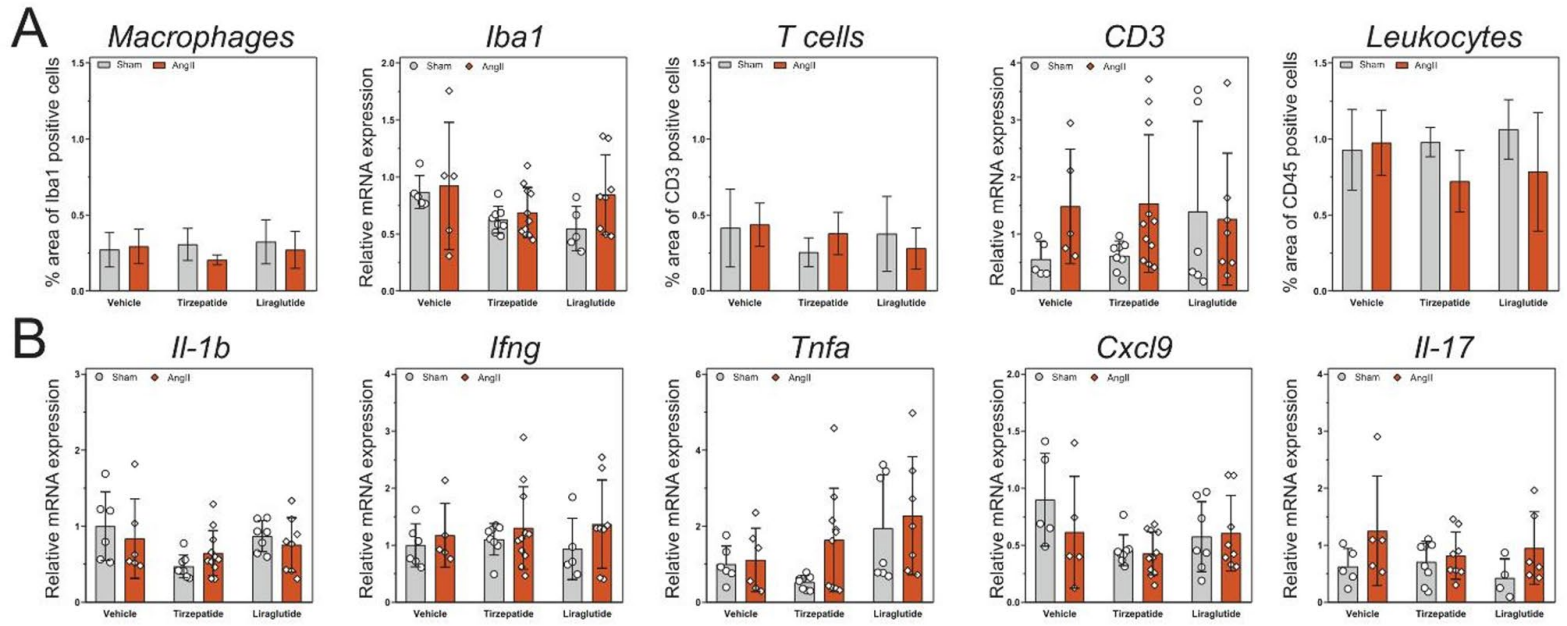
Angiotensin II infusion did not lead to inflammation at the tissue level of the heart. Characterization of immune cell infiltration in the heart with immunohistochemistry and qRT-PCR **A** Expression level of inflammatory markers in the heart **B** Two-way ANOVA followed by Holm–Sidak post-hoc test

Tissue level of inflammation in the heart was not observed. The infiltration of immune cells, such as macrophages (Iba1), T cells (CD3), and leukocytes (CD45) was measured via IHC and qRT-PCR and did not show any significant changes between the groups (Fig. 9A). Furthermore, the expression levels of several inflammation markers (i.e. *Il1b*, *Ifng*, *Tnfa*, *Cxcl9* as well as *Il17*) have not shown any major difference either (Fig. 9B).

## Discussion

In this study, we aimed to investigate the effects of the novel GLP-1/GIP co-agonist tirzepatide and a well-established GLP-1 analogue liraglutide in heart failure. To this end, we used an angiotensin II-induced cardiac injury model in Balb/c mice, a model previously characterised by our group [16]. We show that the novel, dual incretin analogue tirzepatide markedly improves while the GLP- 1R agonist liraglutide only preserves cardiac function and attenuates remodelling in an angiotensin-II infusion-induced cardiac injury model in mice.

As numerous data suggests, incretin analogues have beneficial effects on a wide spectrum of cardiometabolic diseases, including type 2 diabetes, obesity, and ASCVD. However, their use in heart failure is not fully established yet [3]. Several clinical studies are investigating their efficacy in heart failure with preserved ejection fraction (HFpEF), as this type of HF is more common in obese and T2DM patients [26–28]. Nevertheless, despite the promising results in HFpEF treatment, the use of incretin analogues in patients with HFrEF is controversial. Previously, the FIGHT [29] and LIVE [30] trials have shown no significant changes in adverse risk of heart failure-related outcomes and rehospitalisation. On the contrary, potential harmful effects were suggested [8] one of the possible mechanisms includes increased heart rate associated with liraglutide use [31].

The novel dual incretin analogue tirzepatide was shown to have superior effects compared to GLP-1R agonists in terms of weight loss, and tirzepatide was the first incretin analogue to show improved cardiovascular outcomes in a heart failure trial, however, so far only in patients with HFpEF [32]. Interestingly, in the SUMMIT trial, tirzepatide did not raise the heart rate of patients with HFpEF [11], suggesting that potential harmful effects associated with previous GLP-1RAs could be lacking with tirzepatide. Nevertheless, clinical trials or preclinical studies with head-to-head comparisons are not available to date in this regard.

In our preclinical study, we performed a head-to-head comparison of liraglutide and tirzepatide treatment in an angiotensin II-induced cardiac injury model with impaired cardiac function (as described earlier [16]), overall representing a non-ischemic HFrEF model. Here, tirzepatide massively reduced the mortality associated with angiotensin II-induced cardiac injury compared to the healthy control animals, whereas treatment with liraglutide showed a statistically non-significant decrease in mortality, suggesting potential beneficial effects regarding tirzepatide use. Moreover, systolic and diastolic cardiac function was preserved with both treatments. In our model, angiotensin II induction caused a significant increase in the levels of hypertrophy and fibrosis markers through its hypertensive and consistent pressure overload producing effects. Several studies described earlier that hypertrophy and fibrosis are attenuated by liraglutide in different preclinical models of heart failure (such as TAC [33], HFpEF [24], ischaemic cardiac injury models [34]). Nevertheless, in the case of tirzepatide, data is scarce regarding animal models of cardiac injury, whereas only one paper reported that TZP might have a positive modulatory effect on cardiac fibrosis on AC16 human cell line [35]. In our study, both Lira and TZP treatment augmented the AngII-induced elevated level of both fibrosis (*Ctgf, Col3a1*) and hypertrophy (*Nppa, Nppb*). Interestingly, in a sub-analysis of the SUMMIT trial, tirzepatide decreased left ventricular mass in HFpEF patients [36], suggesting a potentially beneficial effect on cardiac hypertrophy. Moreover, decreased levels of CRP was associated with GLP-1RA treatment in clinical trials [5, 11]. In our study, increased CRP was seen in mice with AngII infusion that was significantly decreased by tirzepatide treatment (in accordance with the results of the SUMMIT trial).

In our experiment, none of the measured inflammation markers showed a marked elevation in mRNA or systemic levels. Nevertheless, based on literature data, the angiotensin II-induced inflammatory reaction has a rapid time course, with inflammatory markers showing a decreasing trend after 7 days, while pro-fibrotic factors became more pronounced at the later stages [37]. Moreover, cardiac fibroblasts express AT1 receptor, which has a high-affinity angiotensin II binding site. Thus, angiotensin II can activate fibroblasts directly and lead to increased collagen synthesis and extracellular matrix deposition independent of immune cell infiltration and activation [38].

The main difference between the mechanisms of action of the two drugs is that liraglutide only acts on the GLP-1 receptor, while tirzepatide is a co-agonist of GIPR next to the GLP-1R with a greater affinity to the GIPR [39]. Both receptors play a role in maintaining normal glucose homeostasis and have a central anorectic effect, which leads to their anti-obesity effect. Because obesity significantly increases the risk for cardiovascular diseases such as heart failure, it could be an indirect cardioprotective action. However, there are differences between the pathways in terms of the potentially cardioprotective mechanism [40]. Importantly, GIP can directly act on cardiomyocytes, as experiments revealed full-length GIPR mRNA transcript expression in all 4 chambers from hearts of adult C57BL/6J male mice, which was also detected in cardiac myocytes isolated from neonatal C57BL/6J male mice, and in the atrial HL-1 cardiac myocyte cell line [41] and GIPR gene expression analysis demonstrates a significantly higher GIPR expression than GLP1R in the human AC16 cardiac cell line [35]. As for the mechanisms, Liu et al*.* found that tirzepatide attenuates lipopolysaccharide-induced left ventricular remodelling and dysfunction by inhibiting the TLR4/NF-kB/NLRP3 pathway [17]. TGF-β is another important factor in the development of cardiac hypertrophy and cardiomyocyte enlargement as a downstream mediator of AngII signalling. GIP suppressed AngII-induced TGF-β expression in vivo (on GIPR−/− mice) and in vitro (on HL-1 mouse cardiomyocytes) in the experiments of Hiromura et al. [42], suggesting that TZP acts on GIPR can cause the same effect. Moreover, recent study of Taktaz et al. [35] on AC 16 human cardiomyocytes demonstrated that treatment with TZP increased the expression and activity of SERCA2 and phosphorylated PLN and decreased the expression of PKA and CAMKII, essential modulators of calcium signalling and cardiac hypertrophy. Since myocardial remodelling plays a crucial role in the development of heart failure, and a critical feature of heart failure is impaired cardiac calcium metabolism, this effect of TZP may be crucial in the cardioprotective mechanism.

Previously, several pre-clinical and clinical studies investigated the weight-reducing capacity of incretin molecules. It was shown that liraglutide can reduce body weight by 5–10% compared to placebo [43]. This follows its physiological effect, which induces post-prandial insulin secretion in a glucose-dependent manner, but also promotes weight reduction through central pathways, as well as by peripheral effects in the gastrointestinal tract and adipose tissue, as GLP-1 receptors have been identified in CNS and enteric nervous system in addition to pancreatic cells [44]. The novel dual GLP-1 and GIP receptor agonist tirzepatide was approved in 2023 by the FDA for the treatment of obesity, as around a 15% weight loss was observed in the tirzepatide treated patients [45]. Obesity is associated with an increased risk for heart failure development [46], and around one-third of patients with HFrEF is obese [47]. Nevertheless, several epidemiological studies and meta-analysis found a paradoxical association between higher BMI and survival rates compared with normal-weight HFrEF patients (called as “obesity paradox” [46, 48]). This association raised potential concerns with weight loss therapy in HFrEF patients [49]. However, in recent studies, the use of alternative anthropometric measures and adjusting for novel prognostic revisited this paradox, as measuring only BMI does not take into consideration many other important factors, such as age, sex, race, the location or amount of body fat relative to muscle mass or the weight of the skeleton [50]. In fact, obesity in HFrEF patients was associated with increased risk of heart failure hospitalisation [8]. In this regard, optimal weight management should be prioritized in HFrEF patients as well. Nevertheless, the effects of incretin therapy on body composition (e.g. increased relative loss of skeletal muscle [51]) should be investigated in this population, as patients with advanced heart failure often suffer from sarcopenia [52]. In our study, Angiotensin II induction led to weight loss due to several physiological mechanisms, likely related to cardiac dysfunction, reduced food intake, and sympathetic nervous system activation among other potential mechanisms [53]. Our results demonstrated that treatment with tirzepatide can further potentiate this weight reduction effect. Even though tirzepatide was associated with better survival, the use of incretin analogues to treat non-obese heart failure patients needs careful consideration due to this drastic weight loss.

## Conclusions

In this study, we performed a comparative study between GLP-1R agonist liraglutide and GIPR/GLP-1R co-agonist tirzepatide in a mouse model of non-ischaemic cardiac injury induced by continuous angiotensin II (AngII) infusion. AngII induction led to significant cardiac fibrosis and hypertrophy and consequently to heart failure with reduced ejection fraction. Both Lira and TZP preserved cardiac function and decreased markers of hypertrophy and fibrosis, whereas TZP also significantly decreased mortality. These promising results help to answer whether incretin analogues have a potentially beneficial or harmful effects in patients with heart failure with reduced ejection fraction, however further investigation needed to understand the exact mechanism behind their supportive effect.

## Supplementary Information

Supplementary Material 1.

## Data Availability

No datasets were generated or analysed during the current study.

## Abbreviations

GLP-1: Glucagon-like peptide 1
GIP: Glucose-dependent insulinotropic polypeptide
GLP-1R: Glucagon-like peptide 1 receptor
GIPR: Glucose-dependent insulinotropic polypeptide receptor
ASCVD: Atherosclerotic cardiovascular disease
HF: Heart failure
HFpEF: Heart failure with preserved ejection fraction
HFrEF: Heart failure with reduced ejection fraction
T2DM: Type 2 diabetes mellitus
EF: Ejection fraction
LVEF: Left ventricular ejection fraction
LVEDV: Left ventricular end-diastolic volume
LVESV: Left ventricular end-systolic volume
LVSV: Left ventricular stroke volume
CO: Cardiac output
CI: Cardiac index
BSA: Body surface area
LVIDs: Left ventricular internal diameter in systole
LVIDd: Left ventricular internal diameter in diastole
LVFS: Left ventricular fractional shortening
LVAWTs: The thickness of the left ventricular anterior wall in systole
LVAWTd: The thickness of the left ventricular anterior wall in diastole
LVPWTs: The thickness of the left ventricular posterior wall in systole
LVPWTd: The thickness of the left ventricular posterior wall in diastole
LV: Left ventricular mass
RWT: Relative wall thickness
LVRi: Left ventricular remodelling index
AngII: Angiotensin II
Veh: Vehiculum
Lira: Liraglutide
TZP: Tirzepatide
ECG: Electrocardiography
